# Intervention Programs to Promote the Quality of Caregiver–Child Interactions in Childcare: A Systematic Literature Review

**DOI:** 10.3390/ijerph182111208

**Published:** 2021-10-25

**Authors:** Benedetta Ragni, Francesca Boldrini, Ilaria Buonomo, Paula Benevene, Teresa Grimaldi Capitello, Carmen Berenguer, Simona De Stasio

**Affiliations:** 1 Department of Human Studies, LUMSA University, 00193 Rome, Italy; b.ragni@lumsa.it (B.R.); f.boldrini@lumsa.it (F.B.); i.buonomo1@lumsa.it (I.B.); p.benevene@lumsa.it (P.B.); 2 Unit of Clinical Psychology, Department of Neuroscience and Neurorehabilitation, Bambino Gesù Children’s Hospital, 00165 Rome, Italy; teresa.grimaldi@opbg.net; 3 Department of Developmental and Educational Psychology, University of Valencia, Avda. Blasco Ibáñez 21, 46010 Valencia, Spain; carmen.berenguer@uv.es

**Keywords:** systematic literature review, childcare quality, child–caregiver interaction, child socio-emotional development

## Abstract

Sensitive caregiver–child interactions appear fundamental throughout childhood, supporting infants’ wellbeing and development not only in a familial context but in professional caregiving as well. The main aim of this review was to examine the existing literature about Early Childhood Education Context (ECEC) intervention studies dedicated to caregiver–child interaction, fostering children’s socioemotional developmental pathways. Studies published between January 2007 and July 2021 were identified in four electronic databases following PRIMSA guidelines. The initial search yielded a total of 342 records. Among them, 48 studies were fully reviewed. Finally, 18 of them met all inclusion criteria and formed the basis for this review. Main factors characterizing implemented programs were recorded (e.g., intervention and sample characteristics, dimensions of the teacher–child interaction targeted by the intervention, outcome variables, main results) in order to frame key elements of ECE intervention programs. Our review points to a range of fundamental issues that should consider to enhance ECEC interventions’ efficacy, supporting children’s socioemotional development and caregiver–child interaction. Reflections and considerations for future research are provided.

## 1. Introduction

It is a widely shared view that supportive and responsive relationships and experiences represent a fundamental component of infants’ wellbeing from the very early stages of human development [1,2,3]. Furthermore, caregiver–child interactions and their quality appear to be essential in supporting cognitive and behavioral development, together with social and emotional growth [4].

This assumption is not limited to the familial context and parenting experience, but it comprehends professional care in early childhood education services [5]. According to the literature, teacher–child interactions represent the most salient component of Early Childhood Education and Care (ECEC) service quality in terms of children’s social-emotional functioning [6]. Professional caregivers determine the quality of young children’s ordinary experiences in childcare by direct “back-and-forth” interactions [7], and by mediating relationships with peers and with the school environment [8]. Through nurturing and supportive relationships, children are sustained in learning, understanding, and regulating their behavior and emotions [4,9]. Children are encouraged by the interaction with the adult caregiver to regulate their emotional response, take part in social interactions, and experience empathy [10]. Furthermore, positive caregiver–child interactions in the early childcare setting are associated with children’s improved cognitive development and reduced behavioral problems in primary school [11,12], and with their environmental adjustment and academic success at school age [13,14].

The Teaching Through Interactions (TTI) Framework theorized by Hamre and Pianta [7], identifies three broad domains of teacher–child interactions which are considered effective in promoting children’s development and learning process, together with their socioemotional skills: (a) emotional support, (b) classroom organization, and (c) instructional support. The dimension of Emotional Support (ES) is associated with consistent, positive, and sensitive relationships between children and teachers. It includes the warmth and respect displayed in teacher–child interactions, the enjoyment shown during learning activities, the positive and negative effects expressed within the group of children, the teachers’ responsivity and sensitivity, and teachers’ flexibility within activities to respect children interests and autonomy. Classroom Organization (CO) refers to adults’ effective capability to manage children’s behavior in the school environment, structuring everyday routines, and sustaining learning processes. Finally, the dimension of Instructional Support (IS), deeply related to academic success, is assessed in terms of the quality of teachers’ feedback or the use of instructional strategies that encourage higher-order thinking [15].

The TTI dimensions have been translated into professional learning and evaluated in several intervention studies to sustain caregiver–child interaction in the early childhood education context [11,16,17]. However, while results from these studies showed improvements in teachers knowledge, skills, and children outcomes, researchers did not reach strong conclusions about the effective elements of caregiver trainings due to heterogeneity in the focus, design, and implementation of these programs [11]. In addition to this, while several programs focusing on improving children’s cognitive school readiness have been designed and implemented, interventions targeting the caregiver–child relationship and children’s socio-emotional development have been less investigated [11]. Over recent decades, policies across The Organization for Economic Co-operation and Development (OECD) countries are beginning to recognize the important role of warm and supportive relationships with teachers, peer engagement, and teachers’ strategies during play and structured group time in fostering children’s socioemotional development [18]. The social and emotional competencies developed in early childhood are extremely important because they act as the foundation for ongoing health, wellbeing, prosocial relationships, and engagement in learning during primary school.

The current review was designed to advance our understanding of key elements to consider when developing new ECE intervention programs. Specifically, the main aim of the current review was to examine existing ECEC intervention studies dedicated to caregiver–child interaction as the core factor in fostering children’s socioemotional developmental pathways. Moreover, according to the Pyramid Model of Fox and colleagues [19], universal Tier 1 interventions will be taken into account using a preventive perspective. These universal intervention programs support nurturing and responsive teacher–child relationships and high-quality supportive environments targeting all children and enable the identification of those who need additional support (Tier 2) and, eventually, more individualized and intensive programs (Tier 3).

## 2. Materials and Methods

In planning, conducting, and reporting on this study, we followed the guidelines from the Preferred Reporting Items for Systematic Reviews and Meta-Analyses (PRISMA) statement [20]. Strong heterogeneity in both the studies themselves and in the authors’ reporting of outcomes, and a lack of detailed statistical information in many studies, precluded a meta-analysis. Therefore, the authors adopted a systematic narrative approach to report the study’s key findings.

First, we conducted a search to identify existing studies on implemented Tier 1 interventions targeting caregiver–child interaction quality or caregiver-child interaction quality and children’s socio-emotional development. Specifically, we were interested in intervention studies whose outcomes were the quality of caregiver–child interaction and/or the socio-emotional development of the child, not only children’s cognitive school readiness. For this reason, the following inclusion criteria were adopted: (a) articles published between January 2007, when the ECEC Network was established, and July 2021; (b) empirical studies in peer-reviewed, English-language scientific journals; (c) studies with samples comprising teachers/childcare providers (center-based, home-based, and preschool) of children aged 0–5 years; (d) Tier 1 intervention studies focusing on caregiver–child interaction quality or caregiver–child interaction and children socio-emotional development. Moreover, these interventions should have been (e) implemented and (f) include caregiver–child interaction quality and/or children socio-emotional development as outcome variables.

The studies were identified via an Internet search of the SCOPUS, WoS, Eric, and PsycINFO electronic databases. We adopted an iterative search strategy with three sets of terms: (“preschool*” OR “childcare” OR “daycare” OR “kindergarten*” OR “center-based care” OR “home-based care” OR “family-based care”) AND (“caregiver*” OR “educator*” OR “teacher*” OR “professional*”) AND (“intervention*” OR “training” OR “program*” OR “staff training” OR “teacher* training” OR “caregiver* training”) AND (“caregiver interaction skill*” OR “teacher* interaction skill*” OR “teacher-child interaction*” OR “teacher-infant interaction*” OR “teacher-toddler interaction*”).

We excluded: (a) intervention studies with samples comprising teachers/childcare providers of children aged >5 years; (b) non implemented and empirical evaluated studies; (c) Tier 2 and Tier 3 studies (focus on at-risk children with behavioral problems) and in general samples including children with developmental issues; (d) grey literature; (e) articles whose full-text could not be accessed.

**Figure 1:**
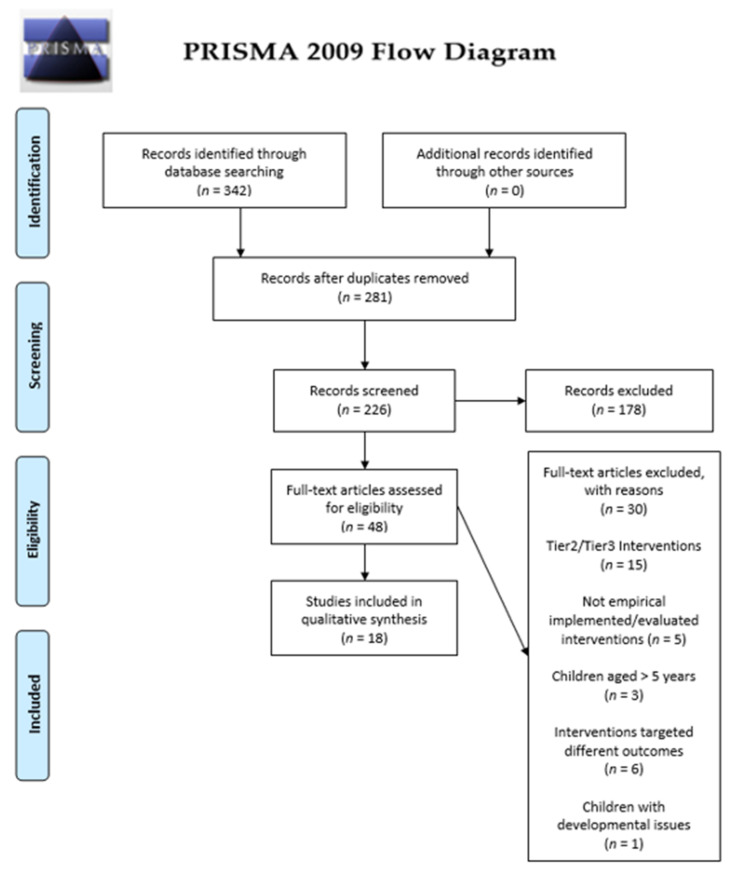
Flowchart for the systematic review procedure.

The flowchart for the systematic review procedure is displayed in Figure 1.

The initial search yielded a total of 342 studies. After eliminating duplicates, 281 remained. Following an initial check of the titles and abstracts, 226 studies were rejected, as they did not meet the inclusion criteria, thus leaving 48 studies to be read thoroughly. Finally, 18 studies met all inclusion criteria and formed the basis for the review.

Detailed information was drawn from each of the relevant articles using a researcher-developed data extraction sheet. The following areas were included: (1) authors, year of publication, and country of data collection; (2) information on the implemented program (name of the program, if it has been validated or not/or if it has been adapted from other validated programs); (3) school characteristics: type of school and type of childcare (center-based childcare or home-base childcare); (4) sample characteristics: number of schools/centers enrolled, number of classes, teachers/caregivers information (number of teachers/caregivers enrolled, gender, average age, education level, years of experience), children information (number of children enrolled, average number of children per classroom, gender, age, SES background, and teacher/child ratio (number of children per teacher) during the intervention implementation; (5) intervention characteristics: TTI dimensions of the teacher–child interaction targeted by the intervention (ES, CO, IS), in-person/web-based intervention, if the intervention includes a group and/or an individual training, activities and duration of training, usage of video and type of video training used (video-modeling or video-feedback), if the intervention included follow-up activities after the intervention, a children curriculum, and a control group; (6) measured variables: outcome variables (evaluated with structured observation or with self-report questionnaires), predictors or covariates, moderators, acceptability/satisfaction/usefulness of the program reported by participants, agreement between teachers and experts evaluations; (7) main results.

The data were coded by three of the authors of this study, and the coding procedure was refined via a consensus discussion procedure. More specifically, the first five articles were randomly chosen for coding. Discrepancies were then resolved via joint review and discussion, and minor adjustments were made to the data extraction sheet. The authors then extracted data from ten articles each, and accuracy was jointly assessed by all three of the author-judges. The information extracted from the set of relevant articles is summarized in Table 1, Table 2, Table 3 and Table 4.

## 3. Results

The 18 studies included in the final review were conducted in 3 countries: The USA (11), The Netherlands (6), and Jamaica (1). All articles had been published between 2009 and 2019.

Eleven studies implemented a validated program, two studies examined an adapted version of a validated program, and in five studies the intervention was applied for the first time.

Nine studies were conducted in preschool sites and the other nine in childcare centers (5 in center-based childcare centers, 3 in home-based childcare centers, and 1 in both center- and home-based centers).

### 3.1. Participants Characteristics

The characteristics of the participants are presented in Table 1.

**Table 1:** Participants characteristics.

Authors (Year), Country	Type of School	No. of Schools	No. Classes	Teachers/Caregivers					Children					Te.-Ch. Ratio
				No.	Sex	Age (Mean)	Education (Degree or Higher)	Years of Experience (Mean)	No.	No. Per Class (Mean)	Sex	Age (Mean)	SES Background	
Baker-Henningham et al. (2009), Jamaica [21]	Preschool	5	27 IG = 15 CG = 12	27 IG = 15 CG = 12	-	-	24	IG = 12 CG = 14	-	21	-	-	Heterogeneous	1:21
Biringen et al. (2012), USA [22]	Childcare	21	-	57 Te.-Ch. pairs IG = 33 CG = 24	-	32	IG = 4; CG = 5	-	57	-	F 40%	IG = 17 mo.; CG = 23 mo.	-	1:1
Driscoll et al. (2011), USA [23]	Preschool	-	-	252 Con.G.= 90; WebG. =96; CG= 66	-	-	83	14	1064 Con.G. 327; WebG. 278; CG 414	14 (enrolled 4 per class.)	F 50.8%	4 y	Low SES (at-risk children)	1:1
Early et al., (2017), USA [6]	Preschool	336	-	486 MMCI = 175; MTP = 151; CG = 160	-	-	91.3%	6	-	19	-	4 y	Heterogeneous	1:9
Fabiano et al. (2013), USA [24]	Preschool	27	-	88 W = 48; I = 40	F 97%	38	34	8	-	23	-	4 y	Low SES (Head Start)	1:23
Fawley et al. (2020), USA [25]	Preschool	1	2	5 Cl. A = 3; Cl. B = 2	F	-	-	-	39	Cl. A = 19; Cl. B = 20	Cl.A 10M, 9F; Cl. B 12M, 8F;	Cl.A = 4.9 y Cl.B= 5 y	Heterogeneous	Cl. A = 3:19 Cl. B = 2:20
Fukkink et al. (2010), the Netherlands [26]	Childcare	2	-	95 IG = 52 CG = 43	-	28	-	5	-	-	-	-	-	1:5–7
Garbacz et al. (2014), USA [27]	Childcare	1	4	12	F	43	41%	11	51	-	F 56%	2–3 y	Heterogeneous	-
Garner et al. (2019), USA [28]	Preschool	3	8 CrC = 5 RC = 3	12	F	-	-	CrC ≤ 1 RC = 2–5	117	-	F 64	4–5 y	Heterogeneous	-
Gray (2015), USA [29]	Childcare (Home-based)	-	-	51 IG = 34 CG = 17	IG: F = 33	44	24%	-	-	-	-	-	-	-
Groeneveld et al. (2011), the Netherlands [30]	Childcare (Home-based)	23	-	49 IG = 24 CG = 25 (only caregiver scoring low on sensitivity)	-	IG = 43; CG = 40	-	-	-	IG = 7 per center CG = 7 per center	-	<4 y	Heterogeneous	-
Groeneveld et al. (2016) the Netherlands [4]	Childcare (Home-based)	23	-	47 IG = 23 CG = 24 (only caregiver scoring low on sensitivity)	-	-	-	-	-	-	-	IG = 27 mo. CG = 25 mo.	Heterogeneous	-
Helmerhost et al. (2017), the Netherlands [8]	Childcare	33	68 IG = 35 CG = 33	139	F	32	7%	8	-	10	-	0–4 y	Heterogeneous	1:5
Jilink et al. (2018), the Netherlands [31]	Preschool	22	-	72 ECE = 17 VIG = 16 ECE + VIG = 18 CG = 21	F 71	46	6%	14	-	-	-	-	Heterogeneous	1:4
Lyon et al. (2009), USA [32]	Preschool	-	4	12	F	37	4	8	78	19–21		3–5 y	Low SES (at-risk children)	-
Moreno et al. (2015), USA [33]	Childcare (Center + Home-based)	-	-	180 EQ = 114 CC = 30; CG = 36	-	EQ = 34 CC = 41 CG = 43	-	EQ = 4 CC = 9 CG = 8	-	-	-	-	-	-
Werner et al., (2018), the Netherlands [5]	Childcare	64	IG = 4 per center CG = 3 per center	64 IG = 34 CG = 30	-	IG = 32 CG = 31	IG = 14% CG = 12%	IG = 4 CG = 4	IG = 66 per center; CG = 61 per center	10	-	0–4 y	Low SES	1:4
Zan and Ritter (2014), USA [34]	Preschool	4	30	60 IG = 38 CG = 22	IG:F = 37 CG:F = 22	IG = 39 CG = 22	IG = 17 CG = 7	IG = 10 CG = 9	-	-	-	-	Low SES (Head Start)	-

Note. CG = control group; Ch. = child/children; Con.G. = consultancy group; F = female; IG = intervention group; MMCI = making the most of classroom interactions; No. = number; Te. = teacher/teachers; WebG.= web group; MTP = my teaching partner; W = workshop; I = intensive; Cl. = classroom; CrC = creative curriculum; RC = responsive classroom; EQ = expanding quality for infants and toddlers; CC = community college course; ECE = early childhood education training; SES = Socioeconomic and Education Status; VIG = video interaction guidance.

The number of enrolled teachers/caregivers ranged from a minimum of 5 to a maximum of 486 (one study did not report the exact number of enrolled caregivers but only the number of caregiver-child pairs). Data on teachers’ gender, age, education level, and years of experience were not reported in all the reviewed studies. From extracted data, it emerged that the majority of enrolled participants were female teachers/caregivers (9/18 studies reported data on gender) with a mean age of 37 years (12/18). The percentage of teachers/caregivers with higher education degrees (bachelor’s degree or higher), instead, ranged from 6% to 91% (12/18) and years of experience from 4 to 14 years (12/18).

With regards to the children, 6 of the 18 studies reported the exact number of children involved (which ranged from 39 to 1064), and 9 included the average number of children per classroom (which ranged from 4 to 21). Only 3 studies reported the exact children’s average age (which ranged from 0 to 5 years). In addition, 9 studies included children from different SES backgrounds while 5 studies examined children with low SES backgrounds only (4 studies did not report information on children’s SES).

Finally, 10 studies reported teacher/child ratio during the implementation of the intervention. In 9 studies, one teacher was identified as interacting with a minimum of 1 and a maximum of 23 children, while in one study, more teachers (2 or 3) implemented the intervention in their classrooms (with 19–20 children).

### 3.2. Intervention Characteristics

Intervention characteristics are summarized in Table 2.

**Table 2:** Interventions’ characteristics.

Authors (Year), Country	Name of the Program	Validate Program	Focus of the Program			In Person/Web-Based	Group Training		Individual Training		Usage of Videos	Follow-Up Activities	Control Group
			ES	CO	IS		Yes/No (Main Activities)	Duration	Yes/No (Main Activities)	Duration	Yes/No (Video Type)	Yes/No (Activities)	Yes/No (Activities)
Baker-Henningham et al. (2009), Jamaica [21]	The Incredible Years Teacher Training program	yes	yes	yes	no	In person	Yes (Psycho-education, role-playing, discussions (applying skills and concepts to their own situations), video)	7 d, once a mo. (over 6 mo.)	Yes (Discuss challenging with the program implementation and potential solutions)	1 h once a mo.	Yes (Video-modeling)	no	Yes (Same Te. resources + experts visit bimonthly)
Biringen et al. (2012), USA [22]	EA-based Intervention in Project Secure Child in Childcare	no	yes	no	no	In person	Yes (Psycho-education, handouts)	2–1 h sessions	Yes (Expert provide written feedbacks on areas of strength and/or of need of improvement)	3–4 visits over 2–3 mo.	Yes (Watch the pretest video with the coach with opportunity to narrate how to improve interactions)	no	Yes (no int.)
Driscoll et al. (2011), USA [23]	Banking Time in MyTeachingPartner Project	yes	yes	no	no	Web-based	no	-	Yes Con.G = materials (books, activities) to implement int. in class. + access website (resources to promote high-quality teaching and te.-ch. relationship); + teaching consultant WebG. = materials + access to the MTP website.	Con.G. = Not specified duration for indivdual training on web; teaching consultant every 2 wk. Web G.: Not specified duration for indivdual training on web	Yes (Video-modeling)	no	Yes (Materials + access to a limited portion of the MTP website)
Early et al., (2017), USA [6]	Making the most of classroom interactions (MMCI) + My teaching partner (MTP)	yes	yes	yes	yes	MMCI = In person MTP = Remote training	MMCI = yes (Psycho-education, discussions, print resources, online library of videos demonstrating best practice) MTP = no	MMCI = 10–2.5 h workshops in 5 d across 5 mo.	MMCI: no MTP: yes (online library of video clips demonstrating best practice + video-feedback and discussion on Te. Interactions with Ch.)	As many feedback-cycles as possible	Yes MMCI = video-modeling MTP = Remote Video-feedback	no	Yes 51 = same online library of video of MMCI and MTP; 109 = 15 h basic professional development course
Fabiano et al. (2013), USA [24]	Professional development in effective classroom management using positive behavioral supports	no	no	yes	no	In person	W = yes (Psycho-education, didactic presentations, discussions) I = yes (Psycho-education, didactic presentations, discussions + experiential training)	W = 6-h I = 6-h + 4 d experiential training	W = no I = yes (feedback session on Te.’s use of techniques)	I = after each practice period	no	Yes I&W = behavioural consultant on class. Observation	no
Fawley et al. (2020), USA [25]	Teacher–Child interaction Training-Universal (TCIT-U)	yes	yes	yes	no	In person	Yes (Psycho-education, discussions, practice worksheets, behaviour-coding, role-playing, videos)	4 h	Yes Consultation: with the psychology Te- reviewed concepts, give and receive feedback and select a target behav. For coaching session; In class. coaching: in-vivo feedback with “bug-in-the-ear” technology” during class.	Consultation: 30 min weekly over 8 wk.; In class coaching: twice-weekly for 10–14 wk., 20 min for each Te.	Yes (Video-modeling)	Yes (booster coaching for 6 wk.)	no
Fukkink et al. (2010), the Netherlands [26]	Video Interaction Guidance for Childcare	yes	yes	no	yes	In person	no	-	Yes (teachers were videotaped while working with their groups + detailed discussion of video clips selected by trainer)	4 sessions	Yes (Video-feedback)	no	Yes (no int.)
Garbacz et al. (2014), USA [27]	Teacher-Child Interaction Training	yes	yes	yes	no	In person	Yes (Workshops, discussions, practice worksheets, role playing, modeling)	9 sessions once a wk. (1.5 h each)	Yes (in class coaching with live coaching and feedbacks)	2 time per wk. over 6–8 wk.	Yes (Video-modeling)	no	no
Garner et al. (2019), USA [28]	Creative Curriculum (CrC) and Responsive Classroom (RC)	yes	yes	no	no	-	-	-	-	-	-	-	no
Gray (2015), USA [29]	Circle of Security-Parenting (COS-P)	yes	yes	no	no	In person	Yes (Discussion, psycho-education, handhouts, videos)	8 wk., 90 min each session	no	-	Yes (Video-modeling)	no	Yes (no int.)
Groeneveld et al. (2011), the Netherlands [30]	VIPP-CC	adapted	yes	yes	no	In person	no	-	Yes (videofeedback)	6 visits	Yes (Video-feedback)	no	Yes (6 phone calls to talk about general developmental topics)
Groeneveld et al. (2016) the Netherlands [4]	VIPP-CC	yes	yes	yes	no	In person	no	-	Yes (videofeedback)	6 visits	Yes Video-feedback	no	Yes (6 calls lasted 15–30 min each to talk about general developmental topics)
Helmerhost et al. (2017), the Netherlands [8]	Caregiver Interaction Profile training	no	yes	yes	yes	In person	Yes (Shared experiences with colleagues)	1 final session	Yes (videofeedback)	4 sessions (each 2 h)	Yes (Video-feedback)	no	Yes (no int.)
Jilink et al. (2018), the Netherlands [31]	Video Interaction Guidance + Early Education Training (ECE)	yes	yes	yes	yes	In person	no	-	Yes ECE = face to face feedback with discussion in class. VIG = Te. Are videotaoed and then shared sessions of video-feedback with the coach	ECE = 9- 2.5 h sessions + 2 biannual meetings 2.5 h focusing on implementation VIG = 4 sessions in 16 wk. each 30 min	ECE: no VIG: yes (video-feedback)	no	Yes (no int.)
Lyon et al. (2009), USA [32]	Teacher–Child Interaction Training	adapted	yes	yes	no	In person	Yes (Workshops, discussions, practice worksheets, role playing)	9 sessions once a wk. (1.5 h each)	Yes (In class coaching with live coaching and written feedbacks)	1–3 wk. for 20 min over 2–4 wk.	no	-	no
Moreno et al. (2015), USA [33]	Expanding Quality for Infants and Toddlers (EQ)	no	yes	yes	yes	In person	Yes (College coursework, applied exercises, textbook)	48 h	Yes EQ0: no EQ5 = in-class coaching with feedback EQ15 = in-class coaching with feedback	EQ0: - EQ5: 5 h EQ15:15 h	no	no	Yes CC = students of the community college course; CG = no int.
Werner et al., (2018), the Netherlands [5]	VIPP-CC	yes	yes	yes	no	In person	no	-	Yes (Te. Are videotaped and then received video-feedback)	6 intervention visists (1.5 h each 2–4 weeks apart)	Yes (Video-feedback)	no	Yes (6 calls lasted 15 each to talk about general developmental topics + brochure about play materials)
Zan and Ritter (2014), USA [34]	Coaching and Mentoring for Preschool Quality	no	yes	yes	yes	In person	Yes (Workshops, role-playing, videos, discussions)	4 bimontly 3 h workshops;Monthly self-reflection	Yes (video-base self-reflection on own videos usign written guides; + peer coaching with teachers’ assistants; + mentoring with class. Teams)	self-reflection monthly; Peer coaching meetings (20–45 min); Monthly class mentoring, 1 h	Yes (Video-modeling Self-reflection on own videos)	no	Yes (no int.)

Note. Behav. = behaviour; CC = community college course; Ch. = child/children; CO = classroom organization; Con.G. = consultancy group; COS-P = Circle of Security-Parenting; CG = Control Group; d = day/days; CrC = creative curriculum; RC = responsive classroom; ES = emotional support; h = hours; Int. = intervention; IS = instructional support; MMCI = making the most of classroom interactions; Mo. = months; Te. = teacher/teachers; WebG.= web group; MTP = my teaching partner; W = workshop; I = intensive; Class. = classroom; EQ = expanding quality for infants and toddlers; ECE = early childhood education training; min = minutes.; TCIT-U = Teacher–Child interaction Training-Universal; VIG = video interaction guidance; VIPP-CC= Video-feedback intervention to promote Positive Parenting in ChildCare; wk. = weeks.

#### 3.2.1. Teacher–Child Interaction Dimensions Targeted by Interventions

Only 5 interventions targeted all the three TTI dimensions of teacher–child interaction, 8 interventions targeted two dimensions and 5 only one dimension. Specifically, 17 interventions focused on ES, 13 on CO, and 6 on IS.

##### Emotional Support

All the nine interventions implemented in childcare and 8 of the 9 interventions in preschool focused on ES.

The eight preschool interventions included group and/or individual training aimed at: supporting teachers in strengthening children’s social and emotional competencies; developing positive relationships with children; labeling the children’s feelings and emotions; respecting the classroom; receiving children’s initiatives and responsively communicating with children; putting emphasis on children’s interests; conveying enthusiasm when interacting with children; and increasing child prosocial skills and emotion self-regulation abilities.

The nine interventions implemented in childcare were focused on: Enhancing caregivers’ knowledge about attachment behaviors and exploration needs; emotional availability and reflective function; affective attunement to the children’s emotions; sensitive responsiveness; respect for autonomy; and verbal/non-verbal aspects of positive interactions (e.g., turning toward the child, making eye contact, following the child, confirming the reception of the initiatives, allowing the children to take turns, acknowledging the actions and intentions of the child, and, finally, the teacher acknowledges his or her actions and intentions, verbalizing children facial expressions and nonverbal cues).

##### Classroom Organization (CO)

Of the 13 interventions that take into account the CO dimension, six were implemented in childcare centers and seven in preschools.

The CO dimension involves organizing and managing children’s behavior, time, and attention. Training is focused on the behavioral management of children, namely: setting up and monitoring appropriate behavior expectations, preventing and redirecting problematic behaviors when they occur, decreasing and preventing inappropriate behaviors, anger management, enhancing problem-solving skills, using specifically labeled praise to promote a targeted behavior, increasing positive attention for appropriate children behavior, and responding to desirable child behavior.

Furthermore, teachers learn how to set clear classroom rules and routines, to become aware of the importance of a well-organized classroom with sufficient and developmentally appropriate learning materials, to encourage and motivate students by maximizing their engagement, to have an active role in the classroom and to actively contribute to the child’s learning without “taking over” the children’s learning.

Interventions focused on CO and implemented in childcare put more emphasis on inductive discipline as non-coercive responses to difficult child behavior, positive reinforcement (praising the child for positive behavior and ignoring negative attention-seeking behaviors), structuring and setting limits (refers to a caregiver’s ability to communicate expectations toward children clearly and set clear and consistent limits), behavior guidance, facilitation of learning and development, and facilitated exploration and autonomy.

##### Instructional Support (IS)

Six studies implemented training including the IS dimension, specifically 3 with preschool teachers and 3 with childcare caregivers.

Training includes promoting children’s higher-order thinking, providing meaningful feedback to children, and facilitating children’s use of language. Teachers learn to reflect upon their planning, implementation, and evaluation of their instructional activities, while safeguarding the balance between preparing and enriching educational activities on the one hand and ensuring sufficient scope for the personal initiatives of young children on the other.

Childcare caregivers’ training focused on this dimension includes verbal stimulation of children (e.g., teachers are also instructed to label children’s and one’s actions and intentions) and developmental stimulation in general, fostering positive peer interactions (caregiver facilitates, encourages, and stimulates positive interactions between children), quality of verbal feedback, and language modeling.

#### 3.2.2. Interventions Structure

Total of 16 interventions were implemented in person, one in remote (with remote live coaching), and one was web-based (online psychoeducational information and video-modeling without remote live coaching). Nine interventions consist of both a group and an individual training or coaching, three interventions comprise only a group training while seven only an individual one.

In-person group training includes courses and workshops that offer teachers and caregivers different types of activities such as psycho-education on teacher–child interaction skills, role-playing, discussions in order to apply skills and concepts to their own situations, handouts, and practical worksheets. In six studies, the group training also included video-modeling activities (teachers observe videos of high-quality teacher–child interactions), in one study the group training included an experiential training with real children, and one study the training foresaw the use online resources (video clips demonstrating best practice).

With regard to the interventions with individual training (16/18), the activities included are heterogeneous. In 8 of the 9 studies that included both group and individual training, the individual training follows the group one. In one study [21], after group training, teachers can discuss challenges with the program implementation and potential solutions with a consultant. In another study [22], experts provide written feedback on areas of strength and/or which need improvement after the observation of caregiver–child interactions. A different study also provided video-feedback sessions [24]. The intervention by Fawley and colleagues [25] included consultation sessions with the psychology and in-class coaching with in-vivo feedback (with “bug-in-the-ear” technology). Three studies included in-class coaching with feedbacks [27,32,33]. Finally, in one study, after the group training, teachers reflect by themselves on their videos using written guides and this is followed by a peer coaching session with teachers’ assistants and a mentoring with experts [34]. The study of Healmerhost et al. [8] was the only one in which the group session followed the video-feedback individual one.

Of the 7 of the 18 studies with only individual training, only one study provided a group of teachers with self-training and online psychoeducational and video materials, while another group with self-training plus remote video-feedback sessions and discussion on teacher–child interaction with experts [6]. In another study (1/7) the psychoeducational self-training was associated with teaching consultant sessions with experts [23], while in one of the intervention groups of Jilink and colleagues [31].

Face to face feedback with discussion in the classroom was provided. In 5 studies, teachers were videotaped while working with their groups, and then video-feedback sessions were implemented [4,5,26,30,31].

Finally, one study [28] did not report information on the structure of the teachers’ training.

Of the 18 reviewed interventions, 15 use videos during teachers’ and caregivers’ training. Specifically, 6 studies used video-modeling during group training, one video-modeling and self-reflection on own videos, another six video-feedback during individual training, and the final one remote video-feedback sessions. In one study caregivers reviewed their videos with experts and had the opportunity to narrate how to improve their interactions, but experts did not provide verbal feedback on the videos [22].

Only two interventions included follow-up activities, with consultation sessions [24] and booster coaching sessions [25] after the intervention ended.

Five studies did not have a control group.

### 3.3. Measured Variables

Measured variables have been summarized in Table 3.

**Table 3:** Measured variables.

Authors (Year), Country	Measured Variables											
	Outcome Variables								Predictors/Covariates	Moderators	Acceptability/Satisfaction/Usefulness (by Te.)	Agreement Te.-Experts
	Evaluated with Structured Obs.						Evaluated with Self-Report Q. (by Experts)	Evaluated with Self-Report Q. (by Te.)				
	Variables	Pre-Post	Evaluated by	Rating Scale Used								
				Val.	Adap.	Ad-hoc						
Baker-Henningham et al. (2009), Jamaica [21]	Te. positive and negative behav. and commands; Te. promoting Ch. social and emotional competences; Ch. appropriate behav. and level of interest and enthusiasm; Te. provides opportunities for Ch. to share and help each other; Te. warmth	yes	Expert	yes	yes	no	-	Ch. Behav. in class. (ad-hoc Q.); Ch. Behav. perceived to be more difficult (ad-hoc Q.)	-	-	Teacher satisfaction with Int. (Ad-hoc Q.)	-
Biringen et al. (2012), USA [22]	Caregiver-Ch. relational quality (EA); Child’s attachment relevant behav. to the adult; Caregiver’s overall style within the whole class.	yes	Expert	yes	no	no	-	-	-	-	-	-
Driscoll et al. (2011), USA [23]	-	-	-	-	-	-	-	Ch. Language/literacy skills (Q.); Child social-emotional competence (Q.); Ch-Te. Relationship (Q.)	Ch. Characteristic: Scores; Sex; Race; Maternal Education; Class. Characteristics: english proficient; individualized education plan; -. ch. enrolled; family income-to-needs ratio; Te. Characteristics; Adult-centered beliefs about educating children (Q); Self-efficacy (Q.); Advanced degree; Educational background; Support Received (study condition); Minutes on Web; Implementation (yes/-);	-	-	-
Early et al., (2017), USA [6]	Te.-Ch. Interaction (ES,CO,IS)	yes	Expert	yes	no	no	-	Knowledge of effective Te.-Ch. Interactions (Q.); Perceived value of the int. (Q.); Relationship with the coach/instructor (Q.)	-	Te. centered beliefs on educating Ch. (Q.); Coach/Instructor centered beliefs on educating Ch. (adapted Q.); Coach/Instructor k-wledge of effective Te.-Ch- interactions (adapted Q.); Coach/Instructor confidence in their understanding of CLASS tools (Q.);	-	-
Fabiano et al. (2013), USA [24]	Te.-Ch. Interaction (CO) Frequency of ch. and te. behav.	yes	Expert	yes	no	no	-	Overall Class. functioning (Adapted Q.);	-	-	Teacher satisfaction with Int. (Adapted Q.)	-
Fawley et al. (2020), USA [25]	Te. behav.; Child. behav.	yes	Expert	yes	no	no	-	Ch. social and behav. competence (Q.)	-	-	Satisfaction with the training program (Ad-hoc Q.)	-
Fukkink et al. (2010), the Netherlands [26]	Behav. of caregivers; Interactions with Ch.; sensitive responsivity; verbal stimulation; nonverbal interactions’ component (micro-level)	yes	Expert	yes	no	yes	-	Work satisfaction (Q.);	-	-	Perceived competence of teachers (Ad-hoc Q.)	-
Garbacz et al. (2014), USA [27]	Te. Behav.	yes	Expert	no	yes	no	-	Social–emotional strengths and behav. concerns in Ch. (Q.)	-	-	Usefulness of training (Ad-hoc Q.)	-
Garner et al. (2019), USA [28]	Ch. and Te. Behav.	no	Expert	yes	no	no	-	beliefs about guiding ch. social-emotional development (Q.)	Te. Level of education and experience; Curriculum type; Gender composition of Te.-Ch- interactions; Te. Beliefes about guiding children’s social-emotional development	-	-	-
Gray (2015), USA [29]	-	-	-	-	-	-	-	Stress and depressive symptoms (Q.) Self-efficacy and competence in supporting ch. scoioemotional development (Q.) Reflective functioning (Q.)	-		Feedback on intervention efficacy and on their perceptions (Ad-hoc Q.)	-
Groeneveld et al. (2011), the Netherlands [30]	Caregiver Sensitivity Global quality of childcare (quality and quantity of stimulation and support available to a ch.)	yes	Expert	yes	no	no	-	Attitude toward sensitive caregiving and limit setting (Q.);	-		Caregiver feedback (Ad-hoc Q.)	-
Groeneveld et al. (2016) the Netherlands [4]	Ch. Wellbeing (general positive state of the Ch.-the extent to which ch. Fell safe, self-confident, relaxed and enjoy activities);	yes	Expert	yes	no	no	-	-	-	Global quality of childcare (quality and quantity of stimulation and support available to a child) (structured Obs. + rating scale); Caregiver Sensitivity (structured Obs. + rating scale); mo. Spent with trusted caregiver;	-	-
Helmerhost et al. (2017), the Netherlands [8]	Caregiver interaction skills;	yes	Expert	yes	no	no	-	-	Global childcare quality (structured Obs.)	-	-	-
Jilink et al. (2018), the Netherlands [31]	Te. Interactive skills	yes	Expert	yes	no	no	-	-	-	-	-	-
Lyon et al. (2009), USA [32]	Teacher in interactions with Ch.;	yes	Expert	yes	no	no	-	-	-	-	satisfaction with Intervention (Ad-hoc Q.)	-
Moreno et al. (2015), USA [33]	Te. Interaction skills	yes	Expert	yes	no	no	-	Knowledge on infant-toddler development (Ad-hoc Q.); Self-efficacy (Adapted Q.)	Modernity in education practicing with Ch. (Q.); Negative views toward childcare field (Ad-hoc Q.); professional status	-	-	-
Werner et al., (2018), the Netherlands [5]	Cargiver sensitive responsiveness; General childcare quality;	yes	Expert	yes	no	no	-	Attitude toward caregiving (Q.);	Ch. Group size; Caregiver-Ch. Ratio;	-	Intervention Evaluation (Ad-hoc Q.)	-
Zan and Ritter (2014), USA [34]	Te. Interaction skills	yes	Expert	yes	no	no	-	-	Te. Education level	-	-	-

Note. Adap. = adapted; Behav. = behavior; Ch. = child/children; CO = classroom organization; EA = Emotional Availability; Obs. = Observation; ES = emotional support; Int. = intervention; IS = instructional support; Q. = questionnaire; Te. = teacher/teachers; Val. = validated.

#### 3.3.1. Outcome Variables

In 16 studies, experts evaluated teacher/caregiver–child interactions with structured observations, and 15 of these observations were performed both before and after the delivered intervention.

In 11 studies, the evaluation focused on teachers’ skills/behaviors only, while in one study on children’s behaviors/wellbeing only. Only in 6 studies, both teachers and child behaviors were evaluated. In 2 childcare studies, the childcare global quality was also taken into account.

In 16 studies, experts used validated rating scales to score structured observations. Among these, in two studies both validated and adapted or ad-hoc scales, and in 10 studies together with experts observations, teachers filled in self-report questionnaires.

In the two studies that did not include structured observations, only teachers self-report questionnaires were used to measure outcome variables.

None of the studies evaluated the agreement between teachers and experts measures.

#### 3.3.2. Predictors, Covariates, and Moderators

Six of the 18 reviewed studies also measured the role of several predictors and/or covariates variables:

1. Children’s characteristics with regards to their SES background, maternal education level, academic scores [23], and gender [28];
2. Teachers’ characteristics with regards to their level of education and the years of teaching experience [23,28,33,34], beliefs about educating children [23,33], self-efficacy [23], negative views toward childcare field [33], beliefs about guiding children’s social-emotional development [28], and Global Childcare quality (quality and quantity of stimulation and support available to a child) [8].
3. Intervention characteristics in terms of the level of support received by experts during the intervention [23], the effectiveness of the implementation [23], the topic of the intervention [28], the child group size and the teacher/child ratio [5].

In addition to this, two studies also tested the effect of moderator variables in terms of:

1. Teachers’/caregivers’ characteristics: level of education, teaching in metropolitan area/outside, years of experience, teacher/child ratio, beliefs on educating children [6], global quality of childcare (quality and quantity of stimulation and support available to a child) [4], sensitivity [4];
2. Children’s characteristics: months spent with the trusted caregiver [4];
3. Expert coach/instructor’s characteristics: beliefs on educating children, knowledge of effective teacher–child interactions, confidence in their understanding of evaluation tools [6].

Finally, nine studies also reported acceptability, satisfaction, and/or usefulness of the intervention measured with self-report questionnaires filled in by the teachers.

### 3.4. Main Results

The main results are reported in Table 4.

**Table 4:** Main results.

Authors (Year), Country	Results				
	Main Results at Post-Test/Follow-Up		Predictors	Moderators	Acceptability/Satisfaction/Usefulness (by Te.)
	Teachers	Children			
Baker-Henningham et al. (2009), Jamaica [21]	IG > positive Te. Behav.; < negative Te. behav.; = Te. commands; CG > negative Te. behav.; > Te. commands; = Te. positive behav.; > IG Te. warmth than CG; IG Te. Provided > opportunities for children to share and help each other than CG;	IG Ch. > appropriate behav. and interest and enthusiasm than CG; 12/14 IG Te. reported that Ch. behav. improve; Te. reported that Ch. behav.remained the same or got worse.	-	-	positive
Biringen et al. (2012), USA [22]	IG caregiver > structuring over time, CG <; IG caregiver > sensitivity over time, CG <; IG caregiver Supportiveness over time>, CG <; IG caregiver Hostility over time<, CG >; IG caregiver Detachment over time<, CG >;	IG Ch. > Responsive over time, CG <; IG Ch. emotionally secure > over time; CG Ch. =;	-	-	-
Driscoll et al. (2011), USA [23]	Ch. who participated in int. developed closer relationships with their Te. over the course of the school year (Closeness) than Ch. who did not participate;		Scores; Minutes on wesite; Impelementation;	-	-
Early et al., (2017), USA [6]	MMCI Te > ES, IS, than CG; MTP Te. > ES than CG; MMCI Te. Knowledge > MTP Te. And CG; both MMCI and MTP Te. perceived their professional development as more valuable than CG; MTP Te. > positive views of the coach/instructor than MMCI Te.	-	-	MMCI*less education -> ES, CO; MMCI*metropolitan area -> IS; MMCI*coach with > years of experience -> IS; MTP*fewer ch. Per adult -> IS	-
Fabiano et al. (2013), USA [24]	1FU I Te. > behav. management-related procedures and Instructional learning formats than W; 3FU W Te. < praise statements than I e. Consultation < functioning class.	-	-	-	positive I > useful than W;
Fawley et al. (2020), USA [25]	< Te. Structuring behav.; < Te. Negative Talk for Class B;	Te. indicated positive child behav. change: < Behavioral Concerns and > Total Protective Factors;	-	-	Positive
Fukkink et al. (2010), the Netherlands [26]	IG Te. > frequent eye contact.; IG Te. > verbally received the initiatives of Ch.; G Te. > allowed Ch. to take turns; IG teachers responded to the initiatives of Ch. < CG;	-	-	-	Te. > confident in their work
Garbacz et al. (2014), USA [27]	Te. skill use > over the course of the training; Te. > skill use associated with > Ch. > socio-emotionl strenghts and < beahav. Concerns;	> socio-emotionl strenghts and < beahav. Concenrns; > protective factors especially for at-risk Ch.	-	-	Positive
Garner et al. (2019), USA [28]	Te. and Ch. in CrC > negative facial expressions than RC; Te. > social-emotional teaching practices < negative facial emotions and > talk about emotions;	-	Te. and Ch. in CrC > negative facial expressions than RC; Te. > social-emotional teaching practices < negative facial emotions and > talk about emotions; interactions with boys only < Te. facial emotion expression; interactions with girls only > Ch. negative facial expression; Te. > negative facial expression or lack of facial expression was also more likely when Ch. > negative emotion; < Te. social-emotional practices > Ch. negative facial expression; Te. > or negative emotions Ch. facial epression	-	-
Gray (2015), USA [29]	IG Te. > self-efficacy and competence managing ch. challenging behaviors and supporting their socioemotional development	-	-	-	positive
Groeneveld et al. (2011), the Netherlands [30]	Global quality increase in IG; >positive attitude toward caregiving and limit setting than CG;	-	-	-	positive
Groeneveld et al. (2016) the Netherlands [4]	Both IG and CG increased Ch. Wellbeing with time;	-	-	In IG Ch. Wellbeing > when they were more familiar with the caregiver	-
Helmerhost et al. (2017), the Netherlands [8]	IG Te. > sensitive responsiveness, respect for auto-my, verbal communication and fostering positive peer interactions;	-	-	-	-
Jilink et al. (2018), the Netherlands [31]	VIG, ECE, and VIG + ECE Te. showed on average > interactive skills compared to CG Te.; ECE effective for Te. verbal communication and developmental stimulation; VIG effective for Te. interactive skills with regard to fostering peer interactions between children; ECE + VIG effective for Te. verbal communication and fostering peer interactions between ch.	-	-	-	-
Lyon et al. (2009), USA [32]	Great improvement from baseline to first phese of int.; The largest mean behavioral gains were observed in the use of unlabeled praise, which increased from an overall mean of 5% at baseline to 9% post-int.; Te. increased their use of behavioral descriptions, reflections, and labeled praise; Inspection of individual teachers’ data suggested that 10 Te. demonstrated > positive behavior over the course of training.	-	-	-	positive
Moreno et al. (2015), USA [33]	EQ had little effect over time on self-efficacy and k-wledge; EQ15 displayed the most consistent pattern of improvements, specifically in the area of support for language and learning.	-	Modernity -> self-efficacy; professional status -> support for language and learning skills;	-	-
Werner et al., (2018), the Netherlands [5]	>IG Te. sensitive responsiveness; <CG Te. sensitive responsiveness; In IG, structured play situations accounted for > sensitivity over time, while in CG < sensitivity over time; Childcare quality > in both groups; IG > positive attitude towards caregiving and limit setting than CG.	-	-	-	positive and IG > of CG
Zan and Ritter (2014), USA [34]	IG Te.: >behav. Management, Productivity, Quality of Feedback, Language modeling; CG Te.: >Negative Climate and < Student Perspective.	-	Behav. management, Productivit, Quality of Feedback, Language modeling > in both degreed and -n-degreed with very little differences	-	-

Note. Behav. = behavior; CG = control group; Ch. = child/children; Cl. = classroom; CO = classroom organization; CrC = creative curriculum; ECE = early childhood education training; I = intensive; EQ = expanding quality for infants and toddlers; ES = emotional support; FU = Follow Up; IG = intervention group; Int. = intervention; IS = instructional support; MMCI = making the most of classroom interactions; MTP = my teaching partner; RC = responsive classroom; Te. = teacher/teachers; VIG = video interaction guidance; * = interaction term to indicate for moderation.

#### 3.4.1. Effect of Intervention at Post-Test or Follow-Up

Total of 15 studies with pre-post intervention research design found positive improvements in teachers skills/behaviors over time [4,5,6,8,21,22,24,25,26,27,30,31,32,33,34]. In particular, Fabiano et al. [24] found better improvements in teachers who received practical training and consultation and feedback by experts together with a psychoeducational workshop, and Moreno et al. [33] found better improvements in caregivers who received more minutes of in-class coaching with feedbacks. Among these 15 studies, three also found positive improvements in children’s behaviors [21,22,27].

Three studies have a cross-sectional research design. Two of them found improvements in teachers skills/behaviors after the intervention [23,28], while one study did not find any improvement in teachers skills/behaviors but only in the teachers’ perceived self-efficacy and competence in managing children challenging behaviors and supporting their socioemotional development [29].

#### 3.4.2. Significant Predictors, Covariates, and Moderators

The effect of several predicting and moderating variables turned out to be significant. With regard to the predictor variables, children’s characteristics in terms of academic scores [23] and gender (interactions with boys are associated with <teachers facial emotion expression; interactions with girls with >children negative facial expression [28]), teachers’ characteristics in terms of the level of education (little differences [34]), years of experience [33], and intervention characteristics in terms of effective implementation (minutes on website/if teachers implemented at least one session of the intervention with children [23]), and topic of the intervention [28], significantly predict interventions’ outcomes.

Instead, with regards to the moderating variables: teachers’ level of education, teaching in metropolitan areas or outside, years of experience, fewer children per adult [6], and months spent with a trusted caregiver [4] significantly moderate the strength of the interventions’ efficacy over time (from pre- to post-intervention).

In all of the 9 studies, the teachers rated positively the acceptability, satisfaction, and usefulness of the interventions.

## 4. Discussion

The eighteen studies included in this systematic review were somewhat homogeneous in their design. Indeed, the majority of the studies included in-person interventions with a group training followed by an individual one, and included the use of videos. However, they were very heterogeneous with regard to other aspects such as sample size, SES of the sample, focus of the intervention programs, duration and frequency of training sessions, and outcomes measured.

Although this strong heterogeneity, and a lack of detailed statistical information in many studies, precluded us from conducting a meta-analysis, we identified several key elements that should be taken into account in future studies to enhance ECEC interventions’ efficacy.

### 4.1. Participant Characteristics

Considering data from our review, only one study took into account the type of childcare setting, examining its moderation effect in interventions aiming at enhancing the quality of caregiver–child interaction [11]. According to the authors of the study, the intervention results do not differ according to the type of childcare, neither in home-based nor center-based care settings (ibidem). The description given by Burchinal and colleagues [35] classified childcare in three different categories, namely: relative care, home-based, and center-based care. Relative care usually involves a family member different from the parents. Home-based care is provided by someone external from the family, in a home-like environment, where generally only one caregiver takes care of a limited number of children [36]. Center-based childcare and preschool are meant to take care of large groups of children based on age, they foresee multiple caregivers and generally follow a structured schedule. Several studies in the existing literature considered associations between childcare characteristics and children’s developmental outcomes, mainly considering differences between family home-based care and center-based care. In particular, findings mostly reported that children who attend center-based care seem to score higher in cognitive tasks compared to their peers in family home-based care [37,38]. On the other hand, results from studies considering potential associations and differences in children’s socioemotional development in a different type of care settings appear still ill-defined, suggesting a need for further evaluations and considerations [39,40,41].

Among structural features of professional care, the child–caregiver ratio is generally considered the most important, especially for infants and toddlers [42,43]. The greatest part of the studies included in our review specified teacher/child ratio during the implementation of the intervention. Data revealed that the caregiver-child ratio [4] and time spent with trusted caregivers [5] are related to the outcomes of interventions designed to strengthen teacher–child interactions in ECEC. In the past decades, other studies consistent with these results recognized with broad consensus that the size of the group of children is associated with more sensitive, responsive, and warm professional caregivers’ attitudes [44,45,46]. Dimensions of educator–child interaction and children’s wellbeing could benefit from reexamining the actual caregiver–child ratio in ordinary care. In order to obtain greater insight into this theme, experimental manipulation of the child–caregiver ratio in early education contexts is needed [47].

Considering children’s features, most of the studies considered in our review included children from different SES backgrounds. Driscoll et al. [23] considered the “family income-to-needs” as a potential predictive factor in implementing an intervention fostering teacher–child relationship, not detecting statistically significant results. By contrast, a recent study conducted by Walls et al. [48] described familial socioeconomic level as a predictive factor of children’s socioemotional development at kindergarten entry. To the best of our knowledge, no study in the literature took into account the relationship between SES and professional caregiver–child interactions. Given the relevance of the association between family SES and developmental outcomes throughout infancy [49], ECEC services would work to narrow socioeconomic differences with early interventions to support children’s developmental pathways [50].

With regard to other participants’ features, the selected studies underlined that teacher level of education [6,34], years of experience [33], and city area in which they work (metropolitan area vs. outside) [6] are related to intervention results. Specifically, teachers’ level of education [6,34] and years of experience [33] significantly predict interventions’ outcomes, while the city area in which teachers work [6] significantly moderated the strength of interventions’ efficacy over time (from pre- to post-intervention).

These findings are consistent with other studies on the association among teacher features, caregiving at school, and children’s social-emotional development.

Several studies, indeed, showed that teachers with more experience and higher education are better at regulating their emotions, being responsive to children’s needs, and encouraging children in expressing themselves in the classroom, when compared to teachers with less experience and education [51,52,53,54,55]. Far from meaning that more experienced or educated teachers directly lead to better child development (e.g., see [56]), such studies suggest that teachers’ characteristics may have a main role in promoting children’s social and emotional skills. It is likely, indeed, that teacher characteristics’ play a role in a wider system of factors (e.g., children features, school features, social, and cultural conditions) influencing the teacher–child interaction, as well as children’s emotional and social development [57].

Furthermore, research showed that these dimensions are impacted by the city area in which centers are located, so that larger city areas or densely populated areas are characterized by higher teacher emotional regulation, sensitivity, and support [52,53,58]. Kotaman [58] underlined that teachers working in urban compared to rural contexts deal with different degrees of parent involvement and expectations, which, in turn, may influence the regularity with which children attend to the educational context and the meaning children attribute to the teacher and the classroom, as well as teacher motivation. Building on these considerations, it is possible that teachers working in a different area may be differently involved or motivated in the interaction with children and caregiving, with potential effects on children’s social and emotional development.

Despite the reported evidence, meta-analyses on the topic did not consider the impact of teachers’ characteristics on the efficacy and effectiveness of the interventions. Further studies and meta-analytic works may deepen current knowledge on teachers’ role and clarify their role in the effectiveness of interventions on children’s social and emotional skills in the educational context.

### 4.2. Intervention Characteristics

The reviewed studies underlined that effective implementation (minutes on website/if teachers implemented at least one session of the intervention with children [23]) and the topic of the intervention training [28] (if it is more focused on emotional support rather than on classroom organization only) are related to the results of the interventions.

Considering the topic of studied interventions, in terms of TTI teacher–child interaction dimensions that programs targeted, the majority of them were focused on the ES dimension, and only five of them targeted all the three dimensions together (ES, CO, and IS). According to Downer and colleagues [23] it is important not to conceptualize the TTI system in a purely aligned way, examining ES in relation to socioemotional development, CO with self-regulation skills, and IS with academic and cognitive outcomes only [18]. Cross-domain relationships should be taken into account to better understand the bidirectional influence between teachers and children [18]. Future intervention studies designed to enhance socioemotional development in children will benefit from the inclusion of not only of the ES dimension but also of CO and IS practices.

Previous metanalyses highlighted some characteristics of the training programs that seem to be related to outcomes. Egert et al. [17] found that the intensity of CLASS-based trainings (defined as hours spent in group training sessions per month) is negatively associated to ES outcomes. Werner et al. [11] found that programs with individual training were more effective than programs without individual training, while program duration and intensity had not been found to moderate program effectiveness. Fukkink and Lont [16] found the largest effects for programs with a fixed curriculum and programs including fewer trainees. However, the small number of studies, their heterogeneity, and missing data restricted the authors’ exploration of effective components within and between studies. Moreover, these issues did not allow us to analyze further moderating factors such as the intensity or the type of the individual training components (e.g., video-feedback or in-vivo feedback).

The strong heterogeneity and missing data also characterize the small number of studies included in our review. For example, even though most studies included individual training (a key element according to Werner and colleagues’ metanalysis [11]), each program implemented it differently (e.g., video-feedback, experts’ feedback without videos, live feedback in the classroom) thus making it difficult to achieve consistent conclusions.

Further studies with solid research designs are needed in order to clarify the role of different training components in the effectiveness of ECEC interventions.

### 4.3. Measured Variables and Outcomes

Overall, most reported studies (17/18) found positive improvements in teachers’ skills/behaviors but only three of them also obtained positive findings for children’s behaviors. These findings are in line with the recent metanalysis of Werner and colleagues [11], which found that training significantly improved caregiver skills, but the effect sizes were largest at the caregiver level rather than at the classroom and child level. As our review found, the small effect could be related to the small number of studies that measured as outcome variables both teachers’ and children’s skills/behaviors (6/18). Future studies should include both of them in order to provide a solution for this issue.

In addition to this, when studies included outcomes on children, a multi-informant approach to maximize the validity of behavioral assessments should be adopted in order to obtain the most valid research results possible. Our results showed that only two out of six studies which included children outcomes used a multi-informant approach. However, no agreement between experts and teachers’ reports was measured.

The findings of this review should also be interpreted in light of the limitations of our own work. First, we only assessed the English-language literature and may, therefore, have overlooked significant findings reported in other languages. Second, although we aimed to conduct an exhaustive search, a relevant search term may have been omitted and consequently relevant studies may have not been retrieved. Third, although we attempted to screen the retrieved studies thoroughly, it is possible that some salient studies were overlooked. Nonetheless, to the best of our knowledge, this review is the first to systematically review universal interventions to foster caregiver–child interaction quality about children’s socioemotional development.

## 5. Conclusions

In conclusion, this review points up a range of critical issues that future studies should consider to enhance ECEC interventions’ efficacy. On the one hand, the review suggests for future research, the importance of analyzing participant and center characteristics as potential moderators of interventions results. Specifically, among participants’ features, the type of childcare, the children–caregiver ratio, the SES background of families involved, and the sociodemographic background of teachers in terms of years of experience should be taken into account. In addition to this, at the intervention level, training programs should include all TTI teacher–child interaction dimensions to better support children’s socioemotional development. On the other hand, the present review encourages future research to provide a solution for methodological issues. First of all, further studies with solid research designs (e.g., RCT) are needed to clarify the role of different training components in the effectiveness of ECEC interventions (e.g., video-feedback vs. in-vivo feedback; web-mediated training vs. in-person training). Moreover, children’s outcomes variables should be measured to verify and confirm intervention at teachers’, children’s, and classroom levels. In doing this, a multi-informant approach to maximize the validity of behavioral assessments should be adopted to obtain the most valid research results possible, and agreement between experts’ and teachers’ reports should be measured.

Given that the 18 studies reviewed were all conducted in Western countries, it will be interesting to investigate ECEC interventions outcomes in Eastern cultures in the future. Teachers’, children’s, and childcare centers’ features may vary widely across different cultures.

## Data Availability

Not applicable.
